# Pulmonary artery to ascending aorta ratio by echocardiography: A strong predictor for presence and severity of pulmonary hypertension

**DOI:** 10.1371/journal.pone.0235716

**Published:** 2020-07-06

**Authors:** Matthias Schneider, Hong Ran, Anna Maria Pistritto, Christian Gerges, Houtan Heidari, Christian Nitsche, Mario Gerges, Christian Hengstenberg, Julia Mascherbauer, Thomas Binder, Irene Lang, Georg Goliasch

**Affiliations:** 1 Department of Internal Medicine II, Medical University of Vienna, Vienna, Austria; 2 Department of Echocardiography, Nanjing First Hospital, Nanjing Medical University, Nanjing, China; 3 Emergency Department, Division of Cardiology, San Paolo Hospital, Savona, Italy; Medizinische Universitat Graz, AUSTRIA

## Abstract

**Background:**

The pulmonary artery (PA) to ascending aorta diameter ratio (PA:A) has been evaluated in numerous studies analyzing cardiac magnetic resonance (CMR) and computed tomography (CT) data. Previously, no transthoracic echocardiography (TTE) cutoffs have been published. We sought to evaluate (1) the feasibility to image the pulmonary trunk in a prospective cohort, and (2) the ability of PA:A derived by TTE to predict pulmonary hypertension (PH).

**Methods:**

We performed a post-hoc analysis of a prospectively recruited consecutive cohort of patients referred to our tertiary center cardiology department due to suspicion for PH. Invasive hemodynamic assessment and quasi-simultaneous TTE was performed in all participants.

**Results:**

A total of 84 patients were included in the analysis, median age was 70.5 years (IQR 58–75), 46 (55%) were female. The PA was significantly wider in the PH group (28mm vs. 22.5mm, p<0.001) with a resulting median PA:A of 0.84 vs. 0.66 (p<0.001). Both PA diameter (r = 0.524 and r = 0.44, both p<0.001) and PA:A (r = 0.652 and 0.697, both p<0.001) significantly correlated with mPAP and with PVR, respectively. Area under the curve for the detection of PH was 0.853 (95%CI 0.739–0.967, p<0.001).

**Conclusion:**

The PA can be visualized in almost all echocardiographic exams, especially when it is dilated. A view showing the pulmonary trunk should be included in every routine TTE. An increased PA:A should raise suspicion for PH and prompt further evaluation and follow-up examinations of these patients.

## Introduction

Pulmonary hypertension (PH) is a disease with significant morbidity and mortality. The first imaging modality in the diagnostic work-up is transthoracic echocardiography (TTE). Despite significant technological advances in recent years and straight diagnostic algorithms, there are still patients with PH who are missed by TTE [1].

The pulmonary artery to ascending aorta diameter ratio (PA:A) has been evaluated thoroughly in numerous studies analyzing cardiac magnetic resonance (CMR) and computed tomography (CT) data. The ratio was able to detect patients with PH [2–6], it correlated with hemodynamics [7–10], and it was predictive for morbidity [11–13] and mortality [14–17]. Different cutoffs for PA:A have been suggested to identify PH, depending on the methodology (CMR or CT), ranging from 0.83 to >1 [2, 14, 18–21]. Previously, no TTE cutoffs have been published.

Measurement of the diameter of the pulmonary trunk and/or the segmental pulmonary arteries is not included in the regular transthoracic echocardiography protocol in daily clinical practice. No prospective studies have so far evaluated how frequent exact visualization of the pulmonary trunk is possible in TTE.

We sought to evaluate (1) the feasibility to image the pulmonary trunk in a prospective cohort, and (2) the ability of PA:A derived by TTE to predict PH.

## Methods

We performed a post-hoc analysis of a prospectively recruited consecutive cohort of patients referred to our tertiary center cardiology department due to suspicion for pulmonary hypertension.

We included all adult patients with clinically indicated right heart catheterization (RHC) between July 2015 and July 2016. The study was conducted in accordance with the amended Declaration of Helsinki. The ethic committee of the Medical University of Vienna approved the conduct of the study (EK# 2012/2014). All patients gave written informed consent before enrollment. Exclusion criteria were patients <18 years of age.

### Echocardiography

Standard transthoracic echocardiograms (2D, Doppler) were performed in all enrolled patients shortly before invasive hemodynamic assessment with echocardiography systems equipped with 3.5 MHz transducers (Vivid E9, Vivid S70; General Electric Healthcare) according to the recommendations and guidelines by the American Society of Echocardiography and the European Association of Cardiovascular Imaging [22–24].

**Fig 1:**
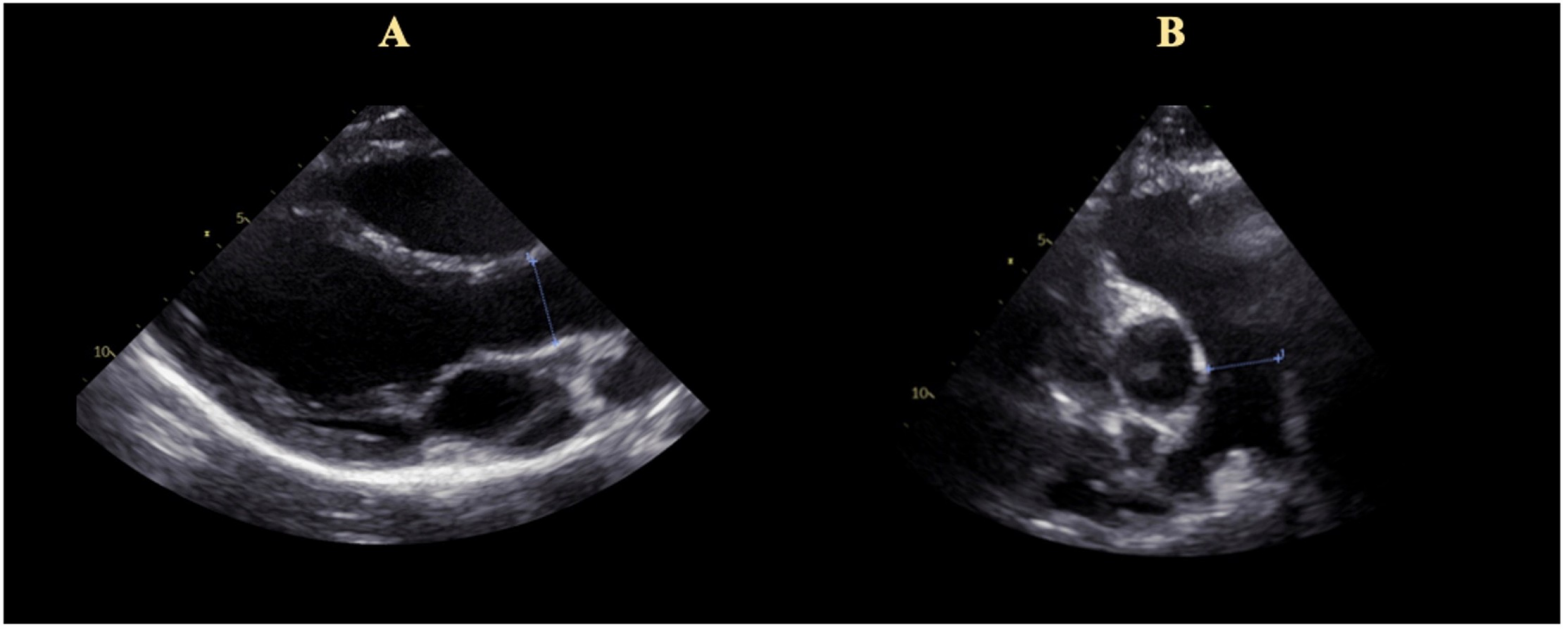
Calculation of the pulmonary artery to ascending aorta ratio (PA:A). Panel A: Parasternal long axis view with measurement of the ascending aorta. Panel B: Parasternal short axis view with visualization of the pulmonary trunk and the bifurcation. Measurement of the main pulmonary artery.

Maximal TR velocity was assessed from different angulations [25]. PA diameter can be measured from different echocardiographic angulations. These include the parasternal long and the parasternal short axis, and the subcostal short axis. The diameter of the ascending aorta was measured in the parasternal long axis view (Fig 1).

### Hemodynamics

Invasive hemodynamic assessment was performed in all study participants. Hemodynamic measurements were performed using a 7F Swan-Ganz catheter (Edwards Lifesciences GmbH, Austria) via a femoral access. Pressures were documented as average of eight measurements over eight consecutive heart cycles using CathCorLX (Siemens AG, Berlin and Munich, Germany). In addition to mean pulmonary arterial wedge pressure (mPAWP), the systolic, diastolic, and mean (mPAP) pulmonary artery (PA) pressures were measured. In a subgroup of patients left ventricular end-diastolic pressure (LVEDP) was measured via left heart catheterization where clinically indicated (suspicion for coronary artery disease, pre-operative assessment of left heart valve disease). Left heart catheterization was performed directly after right heart catheterization.

### Definition of pulmonary hypertension

Presence of PH was defined as a mPAP ≥25 mmHg as suggested by the current guidelines [1]. Additionally, ability to detect mPAP ≥20 mmHg in combination with a pulmonary vascular resistance (PVR) of ≥3 wood units (WU) was tested as recently suggested by the 6^th^ World Symposium on Pulmonary HypertensionTask Force [26].

Elevation of left ventricular filling pressures were defined as mPAWP > 12 mmHg if available, in the remaining patients if LVEDP > 16 mmHg.

### Statistical analysis

Pearson correlation coefficients were calculated to compare invasive with echo measurements. Area under the curve (AUC) and receiver operating characteristic curves (ROC) were performed to evaluate sensitivity and specificity. Differences between two groups were analyzed by T-test analysis. A p value ≤0.05 was considered statistically significant. SPSS Version 24 (IBM SPSS, USA) was used for all analyses.

## Results

A total of 84 patients were included in the final analysis, median age was 70.5 years (IQR 58–75), 46 (55%) were female. Median NT-proBNP was 1,200pg/ml, creatinine 0.98mg/dl, and BUN 18mg/dl. Comorbidities were coronary artery disease in 24 (29%), arterial hypertension in 72 (86%), a history of smoking in 26 (31%), and diabetes mellitus in 16 (19%). Regarding comorbidities, there was no difference between the groups PH and non-PH (Table 1).

**Table 1:** Baseline characteristics, hemodynamic and echocardiographic data.

	Total	PH	No PH	P
	84	70	14	
Female sex, n (%)	46 (55)	37 (54)	9 (64)	0.474
Age, years (median, IQR)	70.5 (58–75)	72 (56–75)	67.5 (59.5–75)	0.582
BSA, m2 (median, IQR)	1.91 (1.73–2.1)	1.91 (1.72–2.1)	1.91 (1.73–2.01)	0.763
Previous medical history				
Coronary artery disease, n (%)	24 (29)	20 (29)	4 (29)	0.793
Diabetes mellitus, n (%)	16 (19)	12 (17)	4 (29)	0.425
Arterial hypertension, n (%)	72 (86)	60 (86)	12 (86)	1
COPD, n (%)	18 (21)	16 (23)	2 (14)	0.21
Hyperlipidemia, n (%)	43 (51)	34 (49)	9 (64)	0.341
History of smoking, n (%)	26 (31)	23 (33)	3 (21)	0.192
Laboratory				
NT-proBNP, pg/ml (median, IQR)	1200 (558–3009)	1229 (593–3009)	901 (320–3434)	0.223
Creatinine, mg/dl (median, IQR)	0.98 (0.8–1.17)	0.97 (0.79–1.17)	1.05 (0.87–1.17)	0.794
BUN, mg/dl (median, IQR)	18 (14–26)	17.6 (13.5–23.8)	24 (16–29)	0.387
Hemodynamics				
mPAP, mmHg (median, IQR)	35.5 (27–47)	38.5 (32–49.5)	20 (17–20)	**<0.001**
PVR, WU (median, IQR)	4.4 (2.1–7)	5 (3–7.5)	1.7 (1.3–2.1)	**<0.001**
mPAWP, mmHg (median, IQR)	12 (8–17)	13 (8–19)	10 (8–12)	**0.038**
LVEDP, mmHg (median, IQR)	12.5 (8–17)	13.5 (7–19.5)	10.5 (7.5–14)	**0.027**
Cardiac index, L/min (median, IQR)	2.7 (2.2–3.2)	2.8 (2.2–3.2)	2.5 (2.25–3.4)	0.894
Echocardiography				
TR Vmax, m/s (median, IQR)	3.6 (3–4.3)	3.9 (3.3–4.4)	2.6 (2.4–2.8)	**<0.001**
TAPSE, mm (median, IQR)	16 (14–19)	16 (14–19)	16 (15–20)	0.991
S‘, m/s (median, IQR)	0.11 (0.09–0.13)	0.10 (0.09–0.12)	0.12 (0.09–0.15)	0.347
RV-GLS, % (median, IQR)	-18 (-13.5-[-24])	-17 (-13-[-24])	-22 (-15-[–26])	0.077
Aorta, mm (median, IQR)	33 (30–36)	33.5 (30–36)	33 (31–37)	0.815
PA, mm (median, IQR)	28 (23–30)	28 (25–31)	22.5 (19.5–26)	**<0.001**
PA:A, median (IQR)	0.8 (0.7–0.94)	0.84 (0.73–0.97)	0.66 (0.61–0.72)	**<0.001**
LV-GLS, % (median, IQR)	-14.5 (-10-[-17])	-15 (-10-[-17])	-13 (-10-[–17])	0.274
LAvolume, ml (median, IQR)	68 (47–93)	68 (48–96)	59 (44–82)	0.13
LAvolume index, ml/m2 (median, IQR)	34 (25–49)	35 (24–51)	31 (25–44)	0.166

PH = pulmonary hypertension, BSA = body surface area, BUN = blood urea nitrogen, mPAP = mean pulmonary artery pressure, PVR = pulmonary vascular resistance, WU = wood units, mPAWP = mean pulmonary capillary wedge pressure, LVED = left ventricular end-diastolic pressure, TR = tricuspid regurgitation, Vmax = maximal velocity, RV = right ventricle, GLS = global longitudinal strain, PA = pulmonary artery, PA:A = pulmonary artery to ascending aorta ratio, LV = left ventricle, LA = left atrium.

All had received quasi-simultaneous right heart catheterization (including LVEDP measurement in n = 62 (74%)) and comprehensive transthoracic echocardiography within a mean of 1 day (±2.8).

Seventy (83%) had pulmonary hypertension, of these 22 (31%) had CTEPH, 30 (43%) had PH due to left heart disease, and 11 (16%) had pulmonary arterial hypertension (Table 2).

**Table 2:** Clinical classification pf pulmonary hypertension in this cohort.

Clinical classification of pulmonary hypertension	
Class 1: Pulmonary arterial hypertension, n (%)	11 (16)
Class 2: PH due to left heart disease, n (%)	30 (43)
Class 3: PH due to lung disease, n (%)	5 (7)
Class 4: Chronic thromboembolic PH, n (%)	22 (31)
Class 5: PH with unclear and/or multifactorial mechanism, n (%)	2 (3)

PH = pulmonary hypertension.

### Hemodynamics

Median mPAP was 38.5mmHg and 20mmHg in the PH group and in the non-PH group. Median PVR was 5 WU and 1.7 WU, cardiac index 2.8 L/min and 2.5 L/min, mPAWP 10mmHg and 13 mmHg, and LVEDP 10.5mmHg and 13.5mmHg, respectively (Table 1).

### Differences between PH and non-PH

There was no difference in TTE parameters of right ventricular function between the PH and the non-PH group. While there was also no difference in diameter of the ascending aorta, the PA was significantly wider in the PH group (28mm vs. 22.5mm, p<0.001) with a resulting difference in median PA:A of 0.84 vs. 0.66 (p<0.001).

Both PA diameter (r = 0.524, p<0.001) and PA:A (r = 0.652, p<0.001) significantly correlated with mPAP. Also, there was significant correlation with PVR for PA diameter (r = 0.44, p<0.001) and for PA:A (r = 0.697, p<0.001) (Table 1).

### PA:A as predictor for the presence of PH

**Fig 2:**
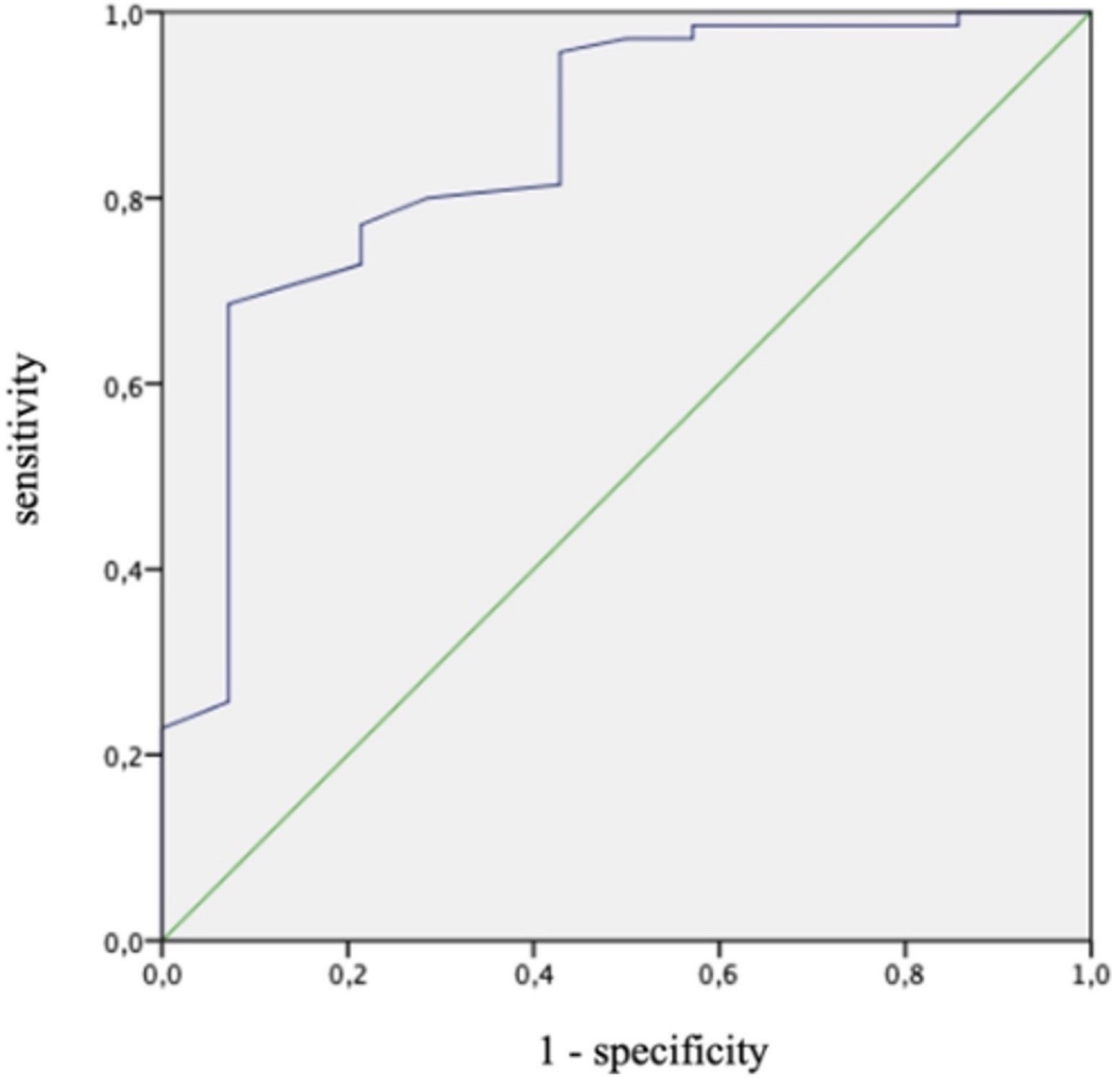
Receiver operating characteristic curve for the detection of pulmonary hypertension (mPAP ≥25mmHg) through PA:A. mPAP = mean pulmonary artery pressure. PA:A = pulmonary artery to ascending aorta ratio.

Area under the curve for the detection of PH (mPAP ≥25mmHg) was 0.853 (95%CI 0.739–0.967, p<0.001). Youden index revealed a PA:A of 0.76 as a cutoff with a sensitivity of 68.6% and a specificity of 92.9% (Fig 2).

Area under the curve for the detection of PH (mPAP ≥20mmHg and PVR ≥3WU) was 0.813 (95%CI 0.684–0.942, p = 0.001). For the cutoff 0.76, sensitivity was 70.8% and specificity was 75%.

## Discussion

Two conclusions can be drawn from our data: (1) the pulmonary trunk can be visualized in most patients, especially when dilated due to the presence of pulmonary hypertension, and (2) an elevated PA:A is strongly predictive for the presence of pulmonary hypertension.

**Fig 3:**
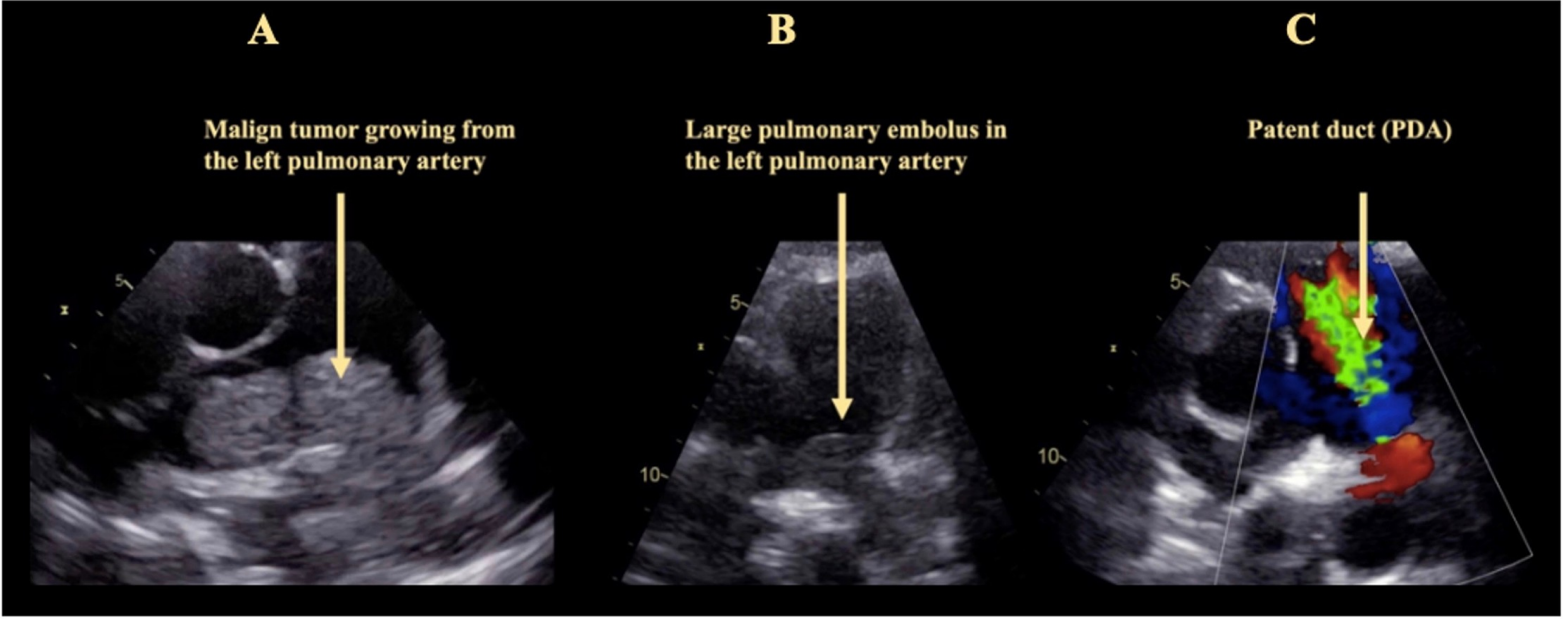
Rare findings that can be diagnosed if the pulmonary trunk is visualized routinely in transthoracic echocardiography. Panel A: A large malign tumor growing from the left pulmonary artery. Panel B: Large pulmonary embolus in the left pulmonary artery. Panel C: Patent duct.

With the 2015 guidelines, a straight forward diagnostic algorithm for how to stratify the probability for PH via TTE was proposed [1]. With TR-Vmax in the center of this algorithm, secondary TTE signs are also to be taken into account. While PA diameter is one of the proposed secondary signs, PA:A is not. With our data, we could show that this ratio can easily be documented in most patients, and that it correlates well with presence and severity of PH. This confirms previous data derived from CT and CMR imaging [2–6]. As of now, measurement of PA diameter is not part of every routine echocardiographic examination. With our data we could demonstrate that visualization of the PA is possible in most patients (in 100% of the patients prospectively imaged in this cohort), either from parasternal or from subcostal angulations. Considering the diagnostic impact shown in this study, and also the possible detection of other underlying diseases such as patent duct, a riding thrombus, or a PA tumor (Fig 3), we propose to include this view into the basic TTE protocol, and the PA:A into the protocol when imaging patients with suspicion for PH.

Recently, a new hemodynamic definition for presence of PH was suggested with mPAP ≥20mmHg in combination with a PVR ≥3WU [26]. Pulmonary artery diameter correlates with mPAP and it can be hypothesized that PA:A primarily identifies PH patients with long-standing severe PH. Though still high with 0.81, area under the curve was lower if those patients with mPAP 20 to 24mmHg were included in our cohort as compared to including only patients with mPAP ≥25mmHg.

There have been numerous studies analyzing the diagnostic value of PA:A derived from CMR and CT calculations. They suggested a ratio of 0.83 to 1.1 for differentiation between those with and without PH and with and without dismal outcome [14–16, 18, 20, 27]. As with other diagnostic tests, PA:A loses sensitivity at the cost of specificity and vice versa depending on the cut-off values chosen. In our data, median PA:A of those without PH was 0.66 (0.61–0.72), of those with PH it was 0.84 (0.73–0.97). A ratio of 0.76 had a sensitivity of 68.6% and a specificity of 92.9% for the detection of PH. Future studies with larger patient cohorts are needed to establish a reliable PA:A that can be applied for TTE.

Interestingly, echocardiography seems to significantly underestimate PA diameter. In a cohort of never-smokers without known heart or lung disease, PA diameter measured by CT was 29mm in men and 27mm in women with a PA:A of 0.9 in both [28]. Another CT study reported a mean PA diameter in the healthy population of 31mm in males and 30mm in females. In a CT-study, PH patients and non-PH patients had PA diameters of 34mm vs. 29mm [16]. A PA diameter of >25mm measured by TTE is considered an indirect sign of PH by the current guidelines [1]. In our TTE cohort, it measured 22.5mm in non-PH and 28mm in PH patients [29]. One transesophageal echocardiographic study evaluated PA and ascending aorta diameters and PA:A. Here, mean PA diameter was 25mm, however mean diameter of the ascending aorta was 25mm, which lead to a PA:A of 1 differentiating between PH and non-PH [30]. More prospective data is needed comparing accuracy of echocardiographic measurements of the pulmonary artery.

Median age of PH patients in this study was 72 years. In a previous study including PH patients with a mean age of 53 years, mean PA diameter measured by TTE was in the same range as in this study [31]. Thus, the PA values reported in the present study for the PA:A cutoff may be representative for the overall PH population. Further prospective research is necessary to validate these findings.

Parameters including more than one variable have the limitation that a measurement mistake must be assumed in each of the assessed variables. This is the strength of solely considering a PA diameter of >25mm as cutoff to detect PH. We therefore suggest to consider the PA:A an additional indirect sign of PH and not a replacement of the assessment of PA diameter.

Considering the increasing distribution of hand-held ultrasound machines where the examiner is limited to 2D imaging and color Doppler imaging without the possibility of CW Doppler measurements, additional 2D echocardiographic signs for hemodynamic assessment such as PA:A can be of great value and should be applied.

## Limitations

The study population was small with only 84 patients. However, considering the complete hemodynamic and echocardiographic assessment, the number nevertheless allows for important insights regarding diagnostic accuracy.

Size of the aorta increases with age. This will lead to a change of PA:A over time independent of changes in pulmonary pressures. As this study only considered one echocardiographic examination in each patient, and patients of different age were included, this could not be corrected for.

Our data reflects the experience of a single tertiary care center. However, the potential advantages of a single-center approach are the enrolment of a homogenous patient population, the adherence to a consistent clinical routine, and a consistent quality of imaging procedures and right heart catheterization.

## Conclusion

The PA can be visualized in almost all echocardiographic exams, especially when it is dilated. A view showing the pulmonary trunk should be included in every routine TTE. An increased PA:A should raise suspicion for PH and prompt further evaluation and follow-up examinations of these patients.

## Supporting information

S1 Data Minimal data set (SPSS file) with age, mPAP, diameter of the aorta (diamAo) and the pulmonary artery (diamPA), and PA:A (PA_to_Ao). (SAV)
